# Cytokinin response factor LcARR11 promotes floral bud physiological differentiation by activating *LcIPT3* and *LcFT1* in litchi

**DOI:** 10.1093/hr/uhaf218

**Published:** 2025-08-26

**Authors:** Xianlin Xie, Hang Zhang, Zishang Kong, Dawei Qian, Xuan Liu, Minglei Zhao, Jianguo Li

**Affiliations:** Key Laboratory of Biology and Genetic Improvement of Horticultural Crops (South China), Ministry of Agriculture and Rural Affairs, Guangdong Litchi Engineering Research Center, College of Horticulture, South China Agricultural University, Guangzhou 510642, China; Key Laboratory of Biology and Genetic Improvement of Horticultural Crops (South China), Ministry of Agriculture and Rural Affairs, Guangdong Litchi Engineering Research Center, College of Horticulture, South China Agricultural University, Guangzhou 510642, China; Key Laboratory of Biology and Genetic Improvement of Horticultural Crops (South China), Ministry of Agriculture and Rural Affairs, Guangdong Litchi Engineering Research Center, College of Horticulture, South China Agricultural University, Guangzhou 510642, China; Key Laboratory of Biology and Genetic Improvement of Horticultural Crops (South China), Ministry of Agriculture and Rural Affairs, Guangdong Litchi Engineering Research Center, College of Horticulture, South China Agricultural University, Guangzhou 510642, China; Key Laboratory of Biology and Genetic Improvement of Horticultural Crops (South China), Ministry of Agriculture and Rural Affairs, Guangdong Litchi Engineering Research Center, College of Horticulture, South China Agricultural University, Guangzhou 510642, China; Key Laboratory of Biology and Genetic Improvement of Horticultural Crops (South China), Ministry of Agriculture and Rural Affairs, Guangdong Litchi Engineering Research Center, College of Horticulture, South China Agricultural University, Guangzhou 510642, China; Key Laboratory of Biology and Genetic Improvement of Horticultural Crops (South China), Ministry of Agriculture and Rural Affairs, Guangdong Litchi Engineering Research Center, College of Horticulture, South China Agricultural University, Guangzhou 510642, China

## Abstract

Cytokinins play crucial roles in regulating the flower bud differentiation in fruit trees. However, the molecular mechanisms by which cytokinins promote flowering in plants are largely unknown. The litchi (*Litchi chinensis* Sonn.) is a typical subtropical fruit tree that suffers from severe alternate fruiting due to unstable flowering. Here, we developed a novel alternate-fruiting management, which can ensure 100% flowering of the on-year trees, while the off-year trees hardly flower at all. The abundance of two types of cytokinins (tZR, iPR) and *LcFT1* expression in the leaves of on-year trees were continuously increased throughout the period of floral bud physiological differentiation. In contrast, these corresponding indicators in off-year trees were maintained at a significantly lower level. Exogenous application of 40 mg/kg 6-BA significantly promoted flowering and increased *LcFT1* expression level in the leaves of the off-year trees. *LcIPT3*, encoding a pivotal rate-limiting enzyme in cytokinin biosynthesis, was identified as the key gene determining the differences in cytokinin levels between on-year trees and off-year trees. Interestingly, we discovered that both *LcIPT3* and *LcFT1* are directly activated by LcARR11, a type-B cytokinin response factor, as demonstrated through both *in vitro* and *in vivo* assays. Furthermore, ectopic expression of *LcARR11* in *Arabidopsis* resulted in elevated *IPT* expression and cytokinin content, alongside increased *FT* expression and earlier flowering. Collectively, our findings suggest that cytokinin-mediated promotion of flowering in litchi might be orchestrated by LcARR11, which could promote floral bud physiological differentiation by activating both *LcIPT3* and *LcFT1*.

## Introduction

Perennial fruit trees often exhibit alternate bearing, a phenomenon marked by high flowering and fruit yield in some years, followed by a sharp decline in both the next year [1, 2]. This pattern creates significant challenges for growers and reduces economic returns. Unstable flowering is a key driver of alternate bearing [1, 3]. Flowering is essential for fruit formation in perennial trees. Both the quantity and quality of flowers directly affect fruit set and development, ultimately determining the tree's yield.

Flowering is a critical phase in plant development, consisting of three stages: floral induction, flower initiation, and floral organ differentiation. Floral induction, the initial step, is regulated by both external environmental cues and internal signals [4]. In *Arabidopsis*, major pathways controlling floral initiation—such as photoperiod, vernalization, gibberellin, temperature, age, autonomic, and hormonal pathways—have been well studied. These pathways often converge on the *FLOWERING LOCUS T* (*FT*) gene, regulating its expression to control flowering time [5]. Floral bud initiation involves two sequential stages: physiological differentiation and morphological differentiation. The florigen protein FT, produced in leaves during physiological differentiation, undergoes phloem-mediated translocation to the shoot apex, forming a functional complex with FD protein, a key bZIP family transcription factor [6]. This complex activates downstream flowering genes, such as *LFY* (*LEAFY*) and *AP1* (*APETALA1*), driving morphological differentiation and the flowering process [7, 8].

Cytokinins are essential plant hormones regulating key processes, including seed sprouting, root apical meristem activity, shoot bud formation, and abiotic stress responses [9]. Their biosynthesis occurs via two pathways: *de novo* synthesis and tRNA degradation, with the former being the primary route. Key enzymes in this process are isopentenyl transferases (IPTs) and lonely guy (LOG), where IPTs act as the rate-limiting enzymes [10]. The principal biologically active cytokinin types include isopentenyl adenine (iP), trans-zeatin (tZ), cis-zeatin (cZ), and dihydrozeatin (DZ), which directly participate in physiological processes. In contrast, trans-zeatin riboside (tZR) and isopentenyladenosine (iPR) are the corresponding riboside forms, typically serving as storage or transport forms that can be rapidly converted into their active counterparts when needed. Generally, tZR and iPR are present at higher concentrations in plants and are relatively stable [11]. Cytokinin signaling resembles the bacterial two-component system (TCS). It involves three core components: *Arabidopsis* histidine kinases (AHKs)**,** histidine phosphotransfer proteins (AHPs)**,** and response regulators (ARRs)**.** When cytokinins bind to AHKs on the endoplasmic reticulum, AHKs autophosphorylate. Then, AHPs act as phosphorelay components that transmit the phosphate signal toward ARRs. ARRs subsequently regulate downstream genes, controlling plant growth and development [12, 13]. The ARR family in *Arabidopsis* is divided into type-A**,** type-B**,** type-C, and APRR genes based on expression patterns, conserved domains, and functions [14, 15]. Type-B ARRs, containing a unique GARP domain, bind DNA and activate downstream targets, including type A ARRs, by interacting with their promoters [16, 17].

Research on woody fruit trees indicates that cytokinins regulate floral bud differentiation. As early as the 1970s, Luckwill suggested cytokinins might influence flower bud formation in deciduous fruit trees [18]. Later studies confirmed that applying cytokinins, such as benzyladenine (BA), significantly increased return bloom in apple [19–21]. In the apple cultivar ‘Tianhong 2’, zeatin riboside (ZR) levels in spur terminal buds declined during physiological differentiation but rose significantly during late morphological differentiation, surpassing levels in vegetative buds [22]. This suggests cytokinins may not drive physiological differentiation but are crucial for morphological differentiation in apple. In pears, foliar application of maleic hydrazide increased cytokinin levels (zeatin, zeatin riboside, and isopentenyl adenine) in lateral buds, potentially enhancing floral bud production [23]. In litchi, cytokinins activity rose during floral bud differentiation, and exogenous cytokinins application mainly promoted the morphological differentiation of floral buds in litchi, i.e. the initiation of flower development, rather than being the primary factor for floral induction [24]. In summary, cytokinins are pivotal regulators of floral bud differentiation in fruit trees. However, their involvement in physiological differentiation remains debated.

Cytokinins also regulate floral bud differentiation in other plants. In *Arabidopsis*, the induction of floral buds correlates with enhanced cytokinin content in leaves and the shoot apex [25]. Exogenous cytokinins can promote flowering in *Arabidopsis* [26, 27]. Similarly, in *Sinapis* (*Sinapis alba* L.), cytokinin levels in the shoot apical meristem increase during flower formation, and cytokinins can induce floral bud development [28, 29]. Bernier proposed that in *Sinapis*, cytokinins and *SaFT* act as independent signals generated in the leaves and then translocated to the shoot apical meristem (SAM). In the SAM, they synergistically regulate flowering: cytokinins drive mitotic activation, while *SaFT* controls the downstream flowering gene *SaSOC1* [30]. In *Arabidopsis*, cytokinin-induced flowering bypasses FT but requires its paralog TSF [27]. In apples, exogenous 6-benzylaminopurine (6-BA) does not activate *MdFT* expression [21]. These findings highlight the need for further research into the molecular mechanisms of cytokinin-mediated flowering.

Litchi originated in Yunnan Province, China, and it is a characteristic fruit tree of tropical and subtropical regions [31]. Adequate winter chilling is essential for litchi floral bud differentiation and successful physiological differentiation is marked by the appearance of ‘whitish millet’ on terminal and axillary buds [32]. However, global warming and frequent warm winters are increasing the difficulty of litchi flowering [33, 34]. Studies have shown that *LcFT1* is crucial for mediating physiological differentiation triggered by low temperatures in litchi, and revealed that genes like *LcSVP9*, *LcSPL3*, and *LcSPL10* regulate litchi floral bud differentiation by modulating *LcFT1* expression [33, 35–37]. However, the molecular mechanisms regulating floral bud differentiation in litchi remain poorly understood. In this study, we developed an innovative alternate-fruiting management for litchi. Following this management, we revealed that on-year trees exhibited markedly elevated cytokinin levels compared to off-year trees. Furthermore, exogenous cytokinin application enhanced *LcFT1* expression in leaves and promoted floral bud physiological differentiation. We further propose that *LcFT1* is directly regulated by the cytokinin response factor LcARR11. Our findings highlight cytokinins’ crucial role in litchi floral bud physiological differentiation and provide new insights into their regulatory mechanisms.

## Results

### Effects of biennial alternate fruiting management on the percentage of flowering shoot of the on-year trees and off-year trees

To explore the regulatory mechanisms that govern floral bud differentiation in litchi, we developed a novel biennial alternate fruiting management. Briefly, the litchi orchard was divided into two zones: one designated as the on-year zone and the other as the off-year zone. These two zones switch to each other every year. For this study, in 2020, the trees in the off-year zone underwent girdling on their trunks when the last flush of autumn shoots matured (Fig. S1). This procedure was intended to inhibit vegetative growth, thereby promoting normal floral bud differentiation and subsequent fruiting in 2021. As a result, the off-year trees in 2020 were switched to on-year trees in 2021. In contrast, in 2020, the trees in the on-year zone were not girdled. Instead, they underwent heavy pruning just as they entered the stage of floral bud physiological differentiation (Fig. S1). During this stage, upright or excessively dense large branches were removed from the base of the trees to stimulate vegetative growth and prevent the normal completion of floral bud differentiation. Consequently, the on-year trees in 2020 were switched to off-year trees in 2021.

As shown in Table 1, with the application of this management in year 2021, among the 200 on-year trees that were randomly selected, 157 trees had a percentage of flowering shoot above 80%, accounting for 78.5%, and 43 trees had a percentage of flowering shoot between 40% and 70%, accounting for 21.5%, no trees had a percentage of flowering shoots below 30%. Subsequently in year 2022, among the 200 on-year trees (which were off-year trees in year 2021), 163 trees had a percentage of flowering shoot above 80%, accounting for 81.5%, and 37 trees had a percentage of flowering shoot between 40% and 70%, accounting for 18.5%, with no trees having a percentage of flowering shoot below 30%. In contrast, the off-year trees did not complete the physiological differentiation of flower buds and produced almost no flowers, with the percentage of flowering shoots among all 200 trees being less than 30% in both 2021 and 2022. These findings indicate that the application of alternate fruiting management can effectively regulate the floral bud differentiation and establish stable on-year and off-year cycles in litchi trees.

**Table 1:** Impact of biennial alternate fruiting management on the percentage of flowering shoot of on-year and off-year trees.

Year	2021			2022		
Percentage of flowering shoot	≤30	40–70	≥80	≤30	40–70	≥80
On-year trees (%)	0	21.5	78.5	0	18.5	81.5
Off-year trees (%)	100	0	0	100	0	0

All trees in the on-year zone exhibit 100% flowering and subsequently produce a large amount of fruit, so they are referred to as on-year trees. In contrast, trees in the off-year zone show flowering rates below 30% and rarely produce fruit, leading them to be classified as off-year trees.

### The relative expression level of LcFT1 in the leaves of on-year trees is significantly higher than that in off-year trees

**Figure 1:**
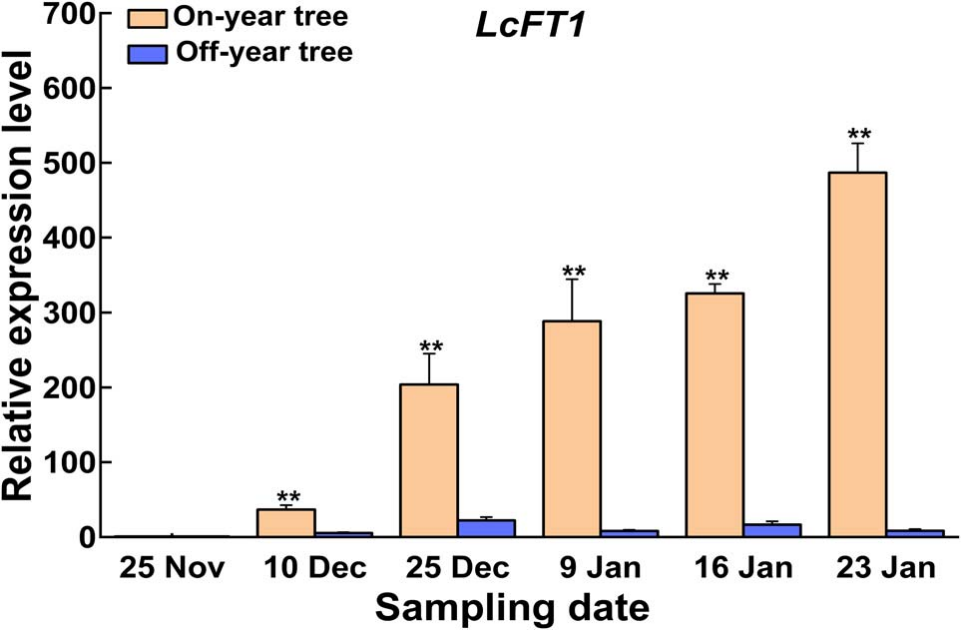
The relative expression level of LcFT1 in the leaves of on-year trees is significantly higher than that in off-year trees. The relative expression levels were calculated based on the expression in off-year leaves sampled on November 25th. The expression level of off-year leaves on 25 November was set as 1, and the relative expression levels of other samples were determined accordingly. Each tree was used as a biological replicate, with a total of three biological replicates, and each sample included three technical replicates. Student’s *t*-test was employed to evaluate significance (**P < 0.01).

Previous studies have shown that LcFT1 is the key FT protein essential for floral bud differentiation in litchi [35], so we collected mature leaves from the terminal shoots of on-year trees and off-year trees to detect the expression of *LcFT1*. The data revealed that the levels of *LcFT1* expression in the leaves of on-year trees continuously increased throughout the period of floral bud physiological differentiation, until it reached the highest value at the ‘whitish millet’ stage on 23 January 2022, whereas the expression of *LcFT1* in the leaves of off-year trees always maintained at a low level (Fig. 1). These findings suggest that the failure of floral bud physiological differentiation in off-year trees may be associated with low expression levels of *LcFT1*.

### Cytokinin content in the leaves of on-year trees is significantly higher than that in off-year trees

**Figure 2:**
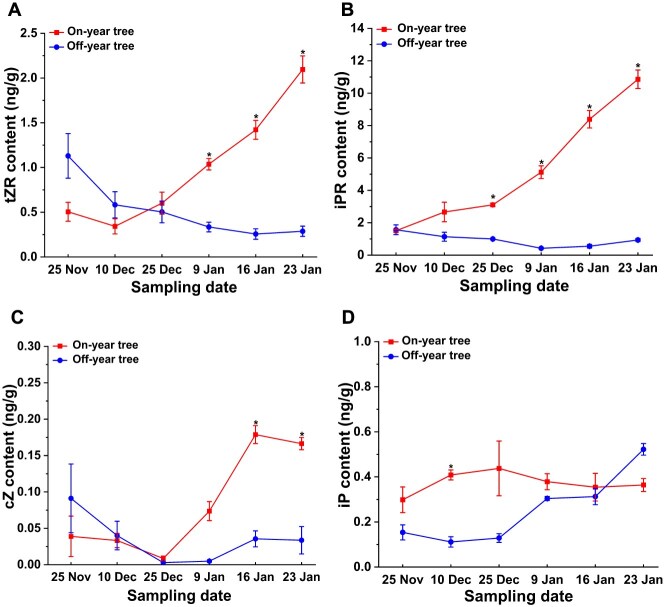
Cytokinin content in the leaves of on-year trees is significantly higher than that in off-year trees. The content of tZR (A), iPR (B), cZ (C), and iP (D) in leaves of on-year trees and off-year trees during the floral bud physiological differentiation in litchi. Experimental data shown are derived from three biological replicates (mean ± SD). Student’s *t*-test was employed to evaluate significance (*P < 0.05).

To identify whether cytokinin metabolism and signaling is involved in the floral bud differentiation of litchi, we determined the endogenous cytokinin content in the leaves of both on-year and of-year trees using UPLC-MS/MS. The results revealed that, among the cytokinins detected, tZR and iPR exhibited relatively high levels in litchi leaves. In the leaves of on-year trees, the contents of both tZR and iPR were continuously increased throughout the floral bud physiological differentiation phase, and were markedly higher than that in off-year trees which showed a slight decline pattern (Fig. 2A and B). Despite in the later stages of floral bud physiological differentiation, the concentration of cis-Zeatin (cZ) in the leaves of on-year trees was markedly higher than that observed in off-year trees, its overall content remained relatively low, not exceeding 0.2 ng/g (Fig. 2C). Additionally, the isopentenyladenine (iP) content of on-year trees showed little variation throughout the entire period of floral bud physiological differentiation and did not significantly differ from that in off-year trees (Fig. 2D). Together, these findings suggest that the elevated flowering rate observed in on-year trees might be associated with the higher levels of cytokinins in the leaves, particularly tZR and iPR.

### Exogenous application of 6-BA to off-year trees improves the flowering rate

**Figure 3:**
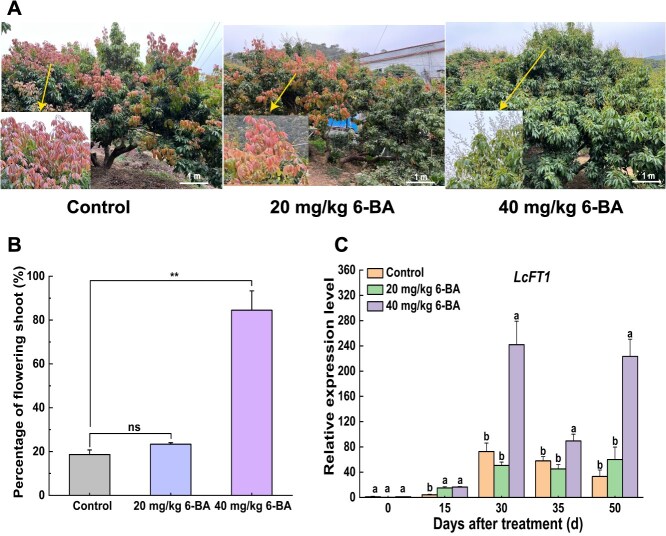
Exogenous application of 6-BA to off-year trees improves the flowering rate. (A) Photographs illustrating the effect of 20 mg/kg 6-BA and 40 mg/kg 6-BA application on shoot growth conditions. The new shootings in both Control and 20 mg/kg 6-BA treatment indicates that the tree has not completed floral bud differentiation and has not entered reproductive growth. Instead, it has entered vegetative growth again. In contrast, the 40 mg/kg treatment induced floral bud differentiation, prompting the tree to enter reproductive growth and produce inflorescences. (B) Quantitative analysis of exogenous 6-BA treatment impacts on flowering rate in off-year trees. Each tree was used as a biological replicate. The experimental data shown are derived from three biological replicates (mean ± SD). (C)The dynamics of *LcFT1* gene expression in leaves after exogenous 6-BA application. Experimental data shown are derived from three biological replicates (mean ± SD), each sample for RT-qPCR was performed with three technical replicates. Statistical analyses employed Student's *t*-test (*P < 0.05, **P < 0.01) or Duncan’s test (dissimilar letters mark significant variations at *P* < 0.05); ‘ns’ represents no significant difference.

To further explore the function of cytokinins in the floral bud physiological differentiation of litchi, we applied exogenous 6-Benzylaminopurine (6-BA) to off-year trees that had just entered the floral bud differentiation phase. For this, we used two different concentration including 20 mg/kg 6-BA and 40 mg/kg 6-BA. 80 days after treatment, the rate of flowering shoot was relatively low in trees treated with 20 mg/kg 6-BA, and most branches produced new shoots. In contrast, treatment with 40 mg/kg 6-BA had a significant effect in promoting flowering, with most branches producing flower spikes (Fig. 3A, Fig. S2). Following the quantitative analysis, we found that the rate of flowering shoot in the off-year trees treated with 20 mg/kg 6-BA displayed no significant difference with that in control, which was 23.4% and 20%, respectively (Fig. 3B). In contrast, the rate of flowering shoot in the off-year trees treated with 40 mg/kg 6-BA reached 80%, which was markedly higher than that in control (Fig. 3B). Furthermore, we examined the expression level of *LcFT1* after 6-BA treatment. The data revealed that application of 40 mg/kg 6-BA resulted in significantly enhanced *LcFT1* expression levels relative to the control, and this upregulation became evident by 30 days after treatment (DAT) and persisted at elevated levels through to the ‘whitish millet’ stage, which occurs at 50 DAT. By contrast, although the relative expression level of *LcFT1* in trees treated with 20 mg/kg 6-BA showed a slight increase at later stages, it was not significantly different from the control and was significantly lower than that in trees treated with 40 mg/kg 6-BA (Fig. 3C). Collectively, these results indicate that cytokinin-mediated promotion of litchi floral bud differentiation is dose-dependent, with 40 mg/kg 6-BA being effective in facilitating the physiological differentiation of floral buds in off-year trees by enhancing the expression of *LcFT1*.

### *LcIPT3* expression is significantly higher in the leaves of on-year trees than that in off-year trees

**Figure 4:**
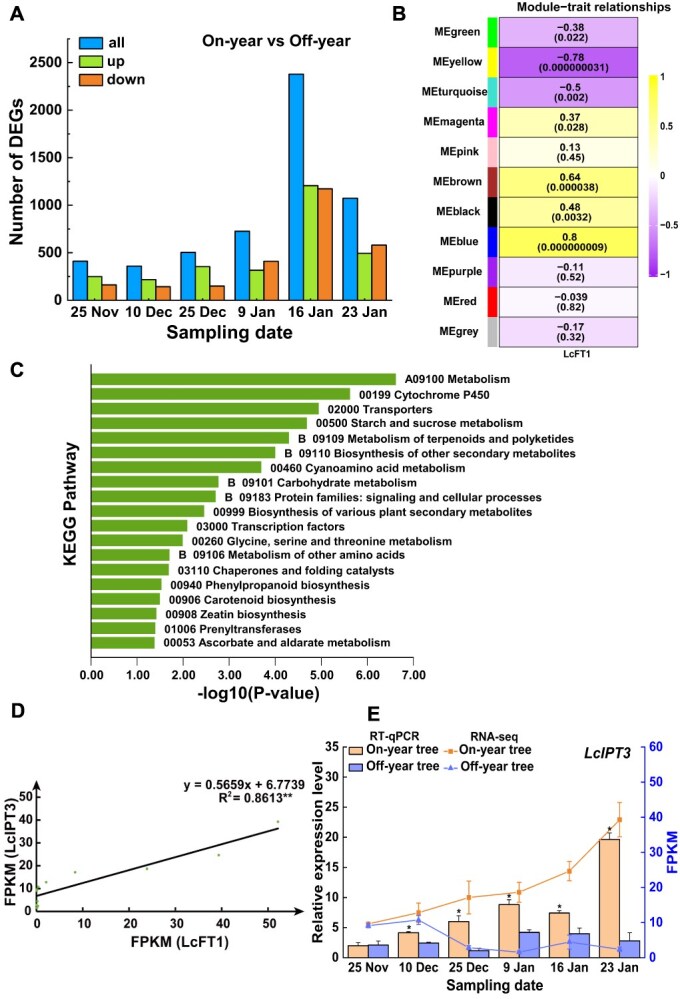
The expression level of *LcIPT3* is significantly higher in the leaves of on-year trees compared to off-year trees. (A) Quantitative distribution of DEGs in leaves between on-year and off-year trees at different sampling periods. (B) The correlation analysis between modules and the relative expression levels of *LcFT1* in samples. Corresponding *P*-values are indicated in parentheses. A positive relationship is indicated by a number greater than 0, while a negative correlation is represented by a number less than 0. The greater the absolute value, the stronger the correlation. (C) KEGG analysis of genes involved in the most relevant module. (D) Correlation analysis of gene expression between LcFT1 and LcIPT3 based on RNA-seq data. (E) Relative expression levels (data from RT-qPCR) and FPKM values (data from RNA-seq) of *LcIPT3* in leaves of on-year trees and off-year trees during the floral bud physiological differentiation in litchi. Relative expression levels are shown in the bar chart, with corresponding FPKM values plotted as line chart. The relative expression levels are presented on the left Y-axis and as normalized FPKM values on the right. Experimental data shown are derived from three biological replicates (mean ± SD), each sample for RT-qPCR was performed with three technical replicates. Student’s *t*-test was employed to assess significance (*P < 0.05, **P < 0.01).

To further reveal the molecular mechanisms underlying the significant differences in flowering rate between on-year and off-year trees, we performed transcriptome sequencing on leaf samples from on-year and off-year trees across six different stages (including 25 November, 10 December, 25 December, 9 January, 16 January, and 23 January). For each stage, three biological replicates were included for both on-year and off-year trees, resulting in a total of 36 samples. FPKM (Fragments Per Kilobase of transcript per Million fragments mapped) was used as the metric to measure transcript or gene expression levels. Subsequently, FPKM values from samples collected at the same time point were compared between on-year and off-year trees. Differentially expressed genes (DEGs) were identified based on the criteria of FDR ≤ 0.01 and a fold change ≥2. For each stage, some DEGs were overlapping, after removing duplicates across all stages, a total of 3602 unique DEGs were obtained (Fig. 4A, Table S1). These DEGs were subsequently subjected to Weighted Gene Co-expression Network Analysis (WGCNA), which organized transcripts into functional modules based on expression profile similarities. Ultimately, the 3602 DEGs were divided into 10 modules, among them, 113 genes that did not belong to any module were excluded (Fig. S3, Table S2). In order to identify potential regulatory genes influencing litchi floral bud differentiation, we performed correlation analysis between the 10 modules constructed from WGCNA and the phenotypic data (the relative expression levels of *LcFT1* obtained from RT-qPCR). Among all identified modules, the blue module demonstrated the highest correlation with *LcFT1* expression (Fig. 4B), and *LcFT1* itself was also classified into the blue module, which contains 673 genes (Table S2). In addition, we conducted KEGG pathway analysis and revealed that 19 pathways were significantly enriched in the 673 DEGs (Fig. 4C). It is of note that the zeatin biosynthesis pathway, including five genes (*LcIPT3*, *LcIPT5*, *LcCKX1*, *LcCYP72A219*, and *LcUGT73C5*), was significantly enriched. Here, we focused on the zeatin biosynthesis pathway for further analysis, given that cytokinin levels are suggested to be associated with the potential for flowering in litchi (Figs 2 and 3).

To identify the key genes involved in zeatin biosynthesis that play a role in the physiological differentiation of floral buds in litchi, we conducted correlation analyses between the FPKM values of these five genes and that of *LcFT1*. The results revealed a strong positive correlation between the expression levels of *LcIPT3* and *LcFT1*, with a correlation coefficient R^2^ of 0.8613 (Fig. 4D). In contrast, the expression patterns of the other four genes did not exhibit a clear trend (Fig. S4). Based on the transcriptomic data, *LcIPT3* showed induced expression that escalated progressively throughout the floral bud physiological differentiation phase in on-year trees. In contrast, *LcIPT3* expression in off-year trees was repressed and remained at a considerably lower level (Fig. 4E). To validate this observation, we conducted RT-qPCR analysis, which revealed a similar expression pattern for *LcIPT3* when compared to the RNA-seq data (Fig. 4E). Together, these findings suggest that LcIPT3 may be the key genes determining the cytokinin content difference between on-year trees and off-year trees.

### Ectopic expression of *LcIPT3* in *Arabidopsis* promotes flowering

**Figure 5:**
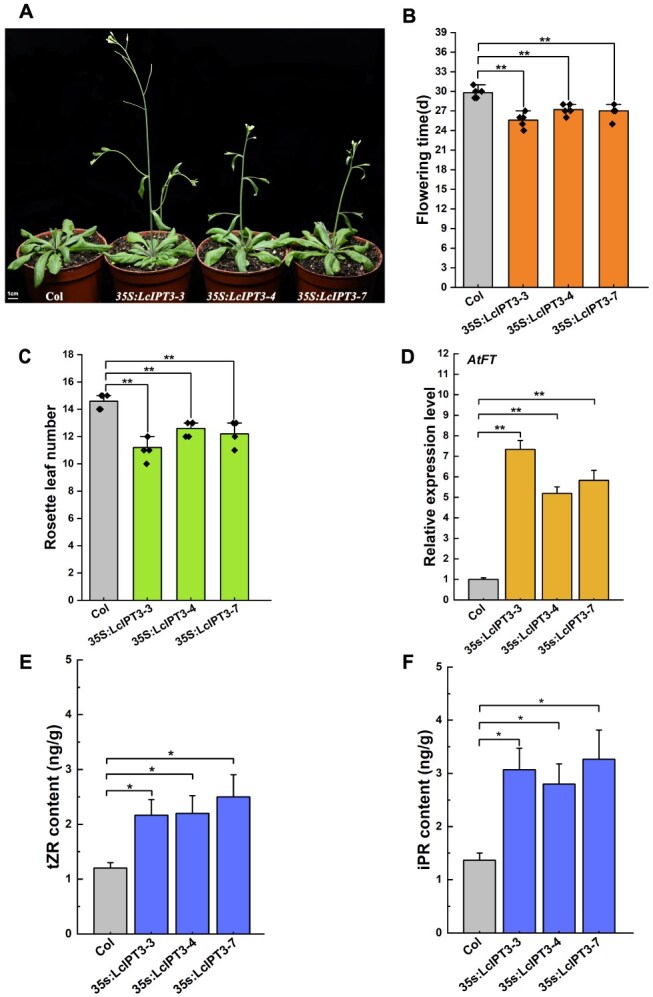
Ectopic expression of *LcIPT3* in *Arabidopsis* promotes flowering. (A) Flowering phenotype of *35S:LcIPT3* transgenic lines in *Arabidopsis*. Flowering time (B) and rosette leaf number (C) in *35S:LcIPT3* transgenic lines and wild-type. The flowering time in the figure refers to the days from seed sowing to the opening of the first flower in *Arabidopsis*. Experimental data shown are derived from five biological replicates (mean ± SD). (D) Comparative analysis of *AtFT* expression levels between *35S:LcIPT3* transgenic lines and wild-type. *AtUBQ* gene was used as the internal reference gene to calculate the expression levels of *AtFT* in the transgenic lines and wild-type plants. The expression level in the wild-type plants was set to 1, and the relative expression levels in the transgenic plants were determined accordingly. Experimental data shown are derived from three biological replicates (mean ± SD), each sample for RT-qPCR was performed with three technical replicates. The contents of tZR (E) and iPR (F) in wild-type and *35S:LcIPT3* transgenic lines. Experimental data shown are derived from three biological replicates (mean ± SD). Student’s *t*-test was employed to assess significance (*P < 0.05, **P < 0.01).

To investigate the function of LcIPT3 in regulating flowering, we constructed a *35S:LcIPT3* overexpression vector and transformed it into wild-type (Col) *Arabidopsis*. Subsequently, for in-depth analysis, we focused on three transgenic lines *35S:LcIPT3-3*, *35S:LcIPT3-4*, and *35S:LcIPT3-7* due to their significantly higher *LcIPT3* expression levels compared to wild-type plants (Fig. S5). As shown in Fig. 5A, under long-day photoperiods, the *LcIPT3*-overexpressing plants exhibited precocious flowering, with floral initiation occurring 2 to 4 days prior to wild-type plants (Fig. 5B), and they produced markedly reduced rosette leaf numbers at the time of flowering relative to the wild-type plants (Fig. 5C). Correspondingly, comparative analysis revealed substantially elevated *AtFT* expression level in *LcIPT3* transgenic lines relative to the wild-type plants (Fig. 5D). Moreover, analysis of endogenous cytokinin content revealed markedly increased levels of iPR and tZR in the *LcIPT3*-overexpressing plants compared to wild-type plants (Fig. 5E and F). Together, these results demonstrate that heterologous expression of *LcIPT3* in *Arabidopsis* could enhance the endogenous cytokinin biosynthesis and promote flowering.

### Transcription factor LcARR11 directly activates *LcFT1* and *LcIPT3* via binding to their promoters

**Figure 6:**
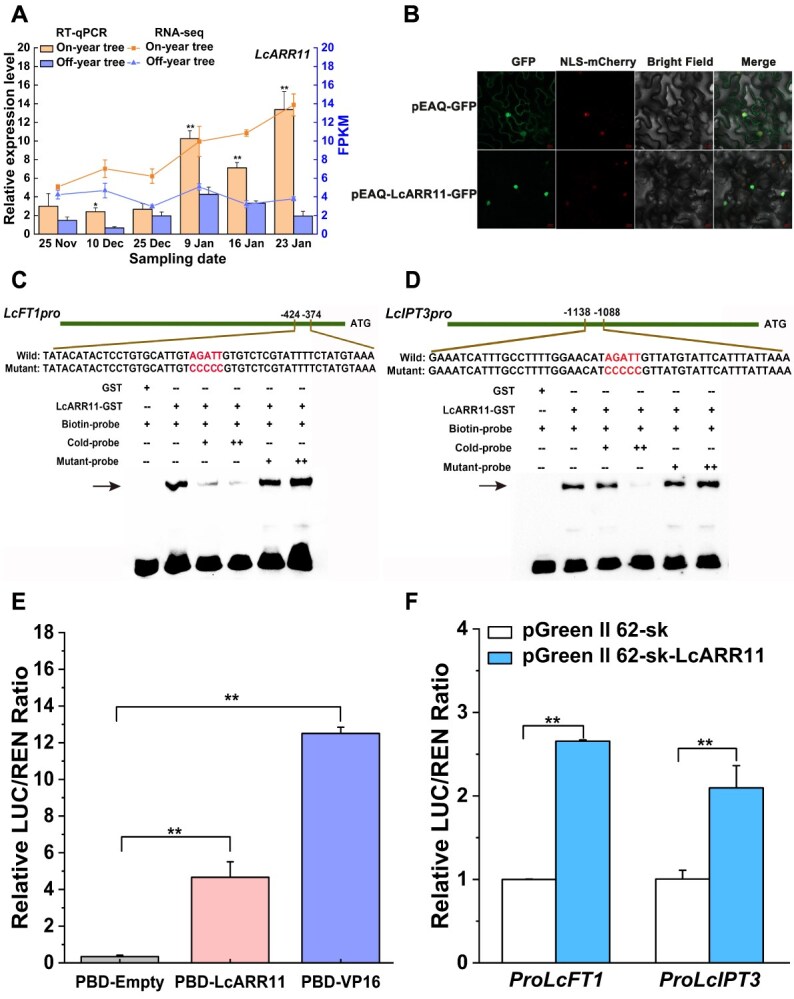
LcARR11 directly activates *LcFT1* and *LcIPT3* via binding to their promoters. (A) Relative expression levels (data from RT-qPCR) and FPKM values (data from RNA-seq) of *LcARR11* in leaves of on-year trees and off-year trees during the floral bud physiological differentiation in litchi. Relative expression levels are shown in the bar chart, with corresponding FPKM values plotted as line chart. The relative expression levels are presented on the left Y-axis and as normalized FPKM values on the right. Experimental data shown are derived from three biological replicates (mean ± SD), each sample for RT-qPCR was performed with three technical replicates. (B) The subcellular localization of LcARR11 in tobacco cells. Fluorescence microscopy was employed to visualize GFP signals in tobacco leaves at 48 h post-infiltration. The nucleus marker (NLS-mCherry) was used as positive control. The composite images illustrate the colocalization of GFP signals with the bright field view. Scale bars represent 20 μm. *In vitro* analysis of LcARR11 interacting with wild-type and mutant promoters of *LcFT1* (C) and *LcIPT3* (D) by EMSA. The nucleotide sequences of wild-type and mutant probes are indicated above. Arrowheads denote migrated bands corresponding to DNA-protein complexes. The symbols ‘+’ and ‘−’ correspond to the presence and absence, respectively, whereas ‘++’ representing the use of a higher concentration of unlabeled or mutant probes for binding competition experiments. GST protein was employed as negative reference. (E) Transcriptional activity of LcARR11 evaluated by dual-luciferase reporter assay through the LUC/REN Ratio. Experimental data shown are derived from six biological replicates (mean ± SD). (F) LcARR11 binds and activates *LcFT1* and *LcIPT3 in vivo*. The effector and reporter constructs were co-introduced into tobacco leaves through *Agrobacterium*-mediated transient transformation. Following 48–72 h of transient expression, transcriptional activation of *LcFT1* and *LcIPT3* promoters by LcARR11 was determined through dual-luciferase (LUC/REN) ratio. Experimental data shown are derived from six biological replicates (mean ± SD). Student’s *t*-test was employed to assess significance (*P < 0.05, **P < 0.01).

To explore how *LcFT1* are transcriptionally regulated during the cytokinin-controlled litchi floral bud physiological differentiation, we first used JASPAR 2022 [38] to predict candidate transcription factors that may interact with the *LcFT1* proximal promoter (Table S3). We then extracted the protein sequences of these transcription factors and subsequently performed BLAST analysis between these transcription factor protein sequences and the protein sequences of all genes in the blue module that was identified through WGCNA analysis (Fig. 4B). This allowed us to screen for transcription factors that could potentially bind to *LcFT1* and whose expression patterns were similar to that of *LcFT1* (Table S4). Using the same method, the transcription factors that could interact with the promoter region of *LcIPT3* were also screened (Tables S5 and S6). Intriguingly, the data suggested that the promoters of *LcIPT3* and *LcFT1* might both be bound by the transcription factor LcARR11, a cytokinin response factor. Subsequently, we performed RT-qPCR and found that the *LcARR11* expression level was increased and markedly higher in the leaves of on-year trees than that in off-year trees during litchi floral bud physiological differentiation, which was similar to the expression patterns of *LcFT1* and *LcIPT3* (Fig. 6A). By constructing a phylogenetic tree, it was found that the LcARR11 protein clusters with AtARR11 in the same evolutionary branch and belonged to the type-B ARR transcription factor family (Fig. S6). Furthermore, subcellular localization analysis indicated exclusive nuclear localization of LcARR11, consistent with molecular characteristics of transcription factor (Fig. 6B).

Previous studies have revealed that type-B ARRs preferentially recognize and bind the GARP *cis*-element (G/A)GAT(T/C) within promoter sequences to modulate downstream gene expression [39–41]. Subsequently, we found that the motif AGATT is present in the promoters of both *LcFT1* and *LcIPT3*. To further verify whether LcARR11 can directly interact with their promoters, we conducted electrophoretic mobility shift assays (EMSA). The results showed that recombinant LcARR11 protein could bind to labeled probes (biotin-probe) containing the *LcFT1* promoter-derived GARP element. While unlabeled competitor (cold-probe) of the same sequence could reduce the binding to the biotin-labelled probes, and labelled mutant probes (mutant-probe) did not affect the binding (Fig. 6C). Similar binding of LcARR11 to the promoter of *LcIPT3* was also observed (Fig. 6D). To further investigate the regulation of *LcFT1* and *LcIPT3* transcriptional activity by LcARR11, we first employed a dual-luciferase assay system in tobacco leaves to determine LcARR11's transcriptional regulatory function. Transient co-expression of pBD-LcARR11 with the reporter construct resulted in marked enhancement of LUC activity (Fig. 6E), indicative of its role as a transcriptional activator. Then, we co-transfected tobacco leaves with constructs containing the *LcFT1* and *LcIPT3* promoters driving the LUC reporter gene (pGreenII 0800-*Pro_LcFT1_*-LUC and pGreenII 0800-*Pro_LcIPT3_*-LUC) along with either the empty vector pGreenII 62-SK or the effector construct pGreenII 62-SK-LcARR11. The results showed that co-expression of the effector pGreenII 62-SK-LcARR11 with the *Pro_LcFT1_*-LUC or *Pro_LcIPT3_*-LUC reporters resulted in a significant increase in the LUC/REN ratio relative to the control (Fig. 6F). Taken together, these results indicate that LcARR11 can activate both *LcFT1* and *LcIPT3* expression via direct binding to their promoters.

### Ectopic expression of *LcARR11* in *Arabidopsis* promotes flowering

**Figure 7:**
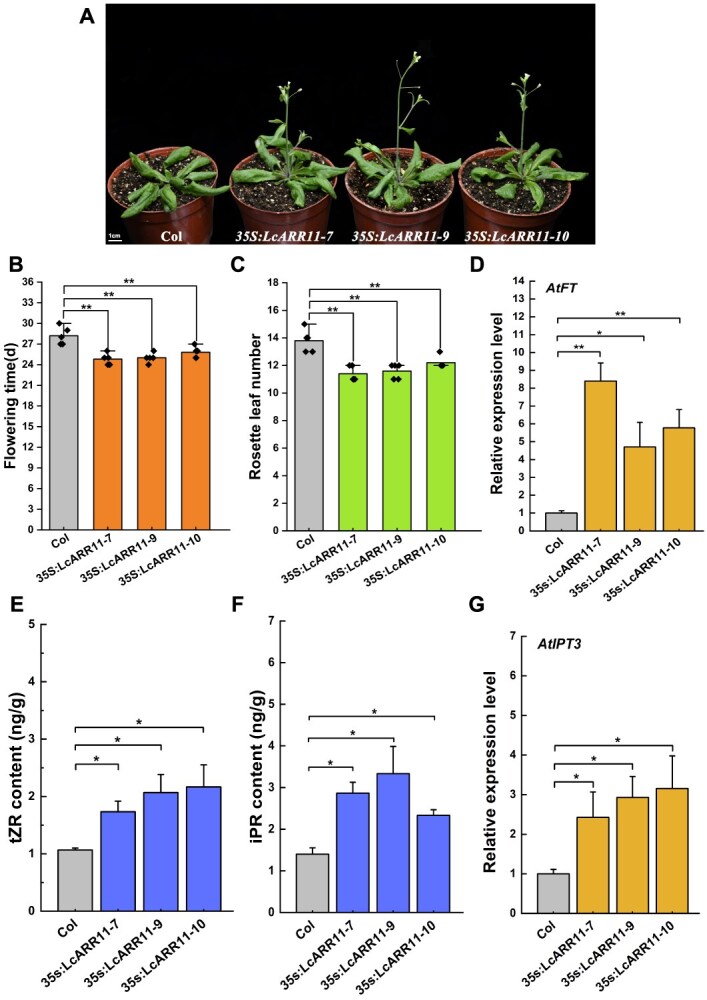
Ectopic expression of *LcARR11* in *Arabidopsis* promotes flowering. (A) Flowering phenotype of *35S:LcARR11* transgenic lines in *Arabidopsis*. Flowering time (B) and rosette leaf number (C) in *35S:LcARR11* transgenic lines and wild-type. The flowering time in the figure refers to the days from seed sowing to the opening of the first flower in *Arabidopsis*. Experimental data shown are derived from five biological replicates (mean ± SD). (D) Comparative analysis of *AtFT* expression levels between *35S:LcARR11* transgenic lines and wild-type. *AtUBQ* gene was used as the internal reference gene to calculate the expression levels of *AtFT* in the transgenic lines and wild-type plants. The expression level in the wild-type plants was set to 1, and the relative expression levels in the transgenic plants were determined accordingly. Experimental data shown are derived from three biological replicates (mean ± SD), each sample for RT-qPCR was performed with three technical replicates. The contents of tZR (E) and iPR (F) in *35S:LcARR11* transgenic lines and wild-type. Experimental data shown are derived from three biological replicates (mean ± SD). (G) Comparative analysis of *AtIPT3* expression levels between *35S:LcARR11* transgenic lines and wild-type. *AtUBQ* gene was used as the internal reference gene to calculate the expression levels of *AtIPT3* in the transgenic lines and wild-type plants. The expression level in the wild-type plants was set to 1, and the relative expression levels in the transgenic plants were determined accordingly. Experimental data shown are derived from three biological replicates (mean ± SD), each sample for RT-qPCR was performed with three technical replicates. Student’s *t*-test was employed to assess significance (*P < 0.05, **P < 0.01).

To validate the role of LcARR11 in regulating floral bud differentiation, the CDS of *LcARR11* was cloned and ligated into the pCAMBIA1302 vector. The plant expression vectors containing *LcARR11* under the CaMV 35S promoter was then transformed into the wild-type *Arabidopsis.* Three transgenic lines *35S:LcARR11-7*, *35S:LcARR11-9*, and *35S:LcARR11-10* with significant expression levels were chosen for further analysis (Fig. S7). We found that the *35S:LcARR11* lines displayed an obvious early-flowering phenotype under long-day conditions (Fig. 7A–C). Additionally, we found that transgenic plants exhibited markedly increased *AtFT* expression relative to the wild-type plants (Fig. 7D). Furthermore, RT-qPCR analysis demonstrated significantly elevated *AtIPT3* expression lever in *LcARR11*-overexpressing lines relative to wild-type plants (Fig. 7G), alongside with this, we also observed that the levels of tZR and iPR in the *LcARR11* transgenic plants were significantly higher than that in the wild-type plants (Fig. 7E and F). Together, these results reveal that heterologous expression of *LcARR11* in *Arabidopsis* could increase the level of endogenous cytokinins and promote flowering probably via enhancing both *AtIPT3* and *AtFT* expression.

## Discussion

Floral bud differentiation in woody fruit trees is tightly regulated by developmental and external cues, such as endogenous hormones, temperature, and humidity [42–44]. However, the mechanisms underlying the regulation of floral bud differentiation in woody fruit trees remain largely unknown. Here, based on a novel cultivation strategy we have developed, we revealed that a transcriptional regulatory module LcARR11-LcFT1/LcIPT3 might mediate the cytokinin-controlled floral bud physiological differentiation in litchi.

### Alternate fruiting management in litchi trees

In general, researchers utilize flowering-related mutants to investigate the mechanisms of flowering regulation. However, this approach is challenging for woody fruit trees due to the difficulty in carrying out genetic assays and the lack of natural resources with flowering defects. On the other hand, the induction of flowering in woody fruit trees is strictly regulated by environmental factors. From this perspective, it seems feasible to promote or inhibit flowering through environmental manipulation, thereby investigating the mechanisms of flowering induction. However, controlling environmental factors such as temperature, light, and humidity in the open field is extremely difficult to achieve, and even if achieved, the cost is prohibitively high. To explore the regulatory mechanisms of flowering induction in litchi trees, we discovered that alternate fruiting management can effectively produce litchi trees with a high flowering percentage. Moreover, it can also generate litchi trees that, to some extent, mimic a mutant with a flowering defect (Table 1). We believe that alternate fruiting management provides a novel model for investigating the regulatory mechanisms of flowering induction in lychee and offers a valuable reference for other woody fruit trees.

### Cytokinins exert a positive influence on the physiological differentiation of floral buds in litchi

Although multiple studies have demonstrated that cytokinins are associated with floral bud differentiation in plants such as apple, pear, litchi, sinapis, and the model plant *Arabidopsis* [23, 24, 27, 29, 45], whether cytokinins are required for the floral bud physiological differentiation remains unclear. Particularly in litchi, a previous study suggested that elevated endogenous cytokinin levels during the process of floral bud differentiation may be linked to floral bud initiation, but they might not be the primary cause [24]. In our study, we measured the cytokinin content in leaves and observed that the levels of tZR and iPR were markedly higher in on-year trees than that in off-year trees. These levels were continuously increased throughout the floral bud physiological differentiation stage and peaked at the ‘whitish millet’ stage (Fig. 2A and B). Furthermore, we found that the contents of tZR and iPR in the leaves showed a strong positive association with the relative expression levels of *LcFT1*, indicating that cytokinins likely serve as key regulators in the floral bud physiological differentiation in litchi. Further this hypothesis gained support from observations that exogenous application of 6-BA to off-year trees increased the flowering rate and enhanced the expression of *LcFT1* (Fig. 3B and C). It is of note that treatment with 20 mg/kg 6-BA had no significant difference compared to the control, but treatment with 40 mg/kg 6-BA had a very noticeable promoting effect on floral bud differentiation (Fig. 3). Therefore, we speculate that the promoting effect of exogenous 6-BA on litchi floral bud differentiation may be dose-dependent. Consistently, previous studies have also shown that the promotive effect of cytokinins on floral induction *in vitro* is highly dependent on dosage, with concentrations exceeding optimal levels may suppress flower formation or cause abnormal floral organ development [46–49].

It is well established that IPTs are the key rate-limiting enzyme genes in the cytokinin biosynthesis process, primarily responsible for the biosynthesis of cytokinins [50]. However, there are fewer reports on the role of IPTs in promoting plant flowering. In this study, by combining RNA-seq analysis, WGCNA, and KEGG pathway analysis, we identified *LcIPT3* as a key gene responsible for cytokinin synthesis involved in floral bud physiological differentiation (Fig. 4E). Overexpression of *LcIPT3* in *Arabidopsis* promoted the accumulation of tZR and iPR in rosette leaves and induced flowering 2 to 4 days earlier than in wild-type plants (Fig. 5). This phenotype closely matches that observed in transgenic *Arabidopsis* overexpressing apple *MdIPT1* [51]. Collectively, our results indicate that *LcIPT3* could contribute to the cytokinin biosynthesis, thereby promoting *LcFT1* expression and the floral bud physiological differentiation in litchi.

### Role of LcARR11 in promoting the physiological differentiation of floral buds by activating *LcIPT3* and *LcFT1* in litchi

The molecular mechanisms by which cytokinins promote flowering remain to be elucidated. Our data revealed markedly elevated *LcFT1* expression in on-year trees relative to off-year trees, with its expression demonstrating strong positive covariation with *LcIPT3* expression pattern (Fig. 4D), indicating that *LcFT1* expression might be regulated by cytokinin signaling. Consistent with this, exogenous 6-BA treatment enhanced the expression of *LcFT1* (Fig. 3C). Supporting our findings, a study in *Arabidopsis* demonstrated that cytokinin-induced flowering involves transcriptional activation of the *TSF* gene, an ortholog of *FT* [27]. Within the cytokinin signaling pathway, type-B ARRs constitute a critical class of transcription factors that can directly interact with the promoters of downstream target genes and activate their expression [52–55]. Interestingly, in our study, by combining JASPAR and WGCNA analyses, we revealed that both *LcFT1* and *LcIPT3* might be regulated by LcARR11, whose expression levels were also markedly higher in on-year trees than in off-year trees (Fig. 6A). Subsequent *in vitro* and *in vivo* experiments confirmed that LcARR11 can specifically bind to the promoters of *LcFT1* and *LcIPT3* and activate their expression (Fig. 6C–F). Moreover, transgenic plants overexpressing *LcARR11* exhibited significantly higher expression levels of *IPT* genes and endogenous cytokinin content in their leaves compared to wild-type *Arabidopsis*, alongside increased *FT* expression (Fig. 7). Earlier work demonstrated that heterologous *RhRR1* expression in *Arabidopsis* could promote early flowering [56]. Collectively, these results support that LcARR11 might play a positive role in promoting floral bud differentiation in litchi by enhancing cytokinin synthesis and *FT* expression.

**Figure 8:**
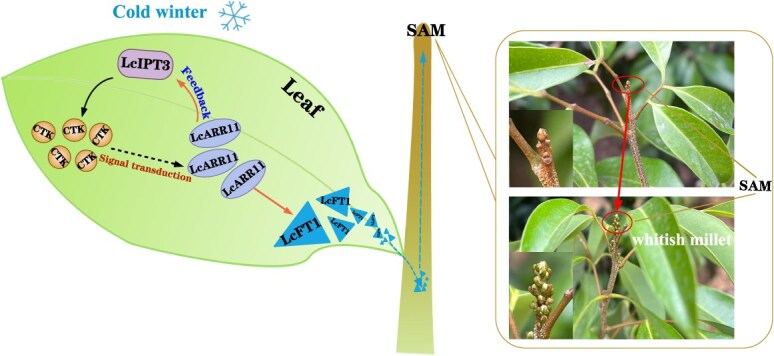
Proposed model of the LcARR11-LcIPT3/LcFT1 regulatory modules in regulating the floral bud physiological differentiation of litchi. As the cold winter season arrives, the expression of *LcIPT3* in leaves gradually rises. This increase enhances cytokinin biosynthesis, which in turn activates the transcription factor LcARR11. Following this, LcARR11 directly promotes the expression of *LcFT1*, facilitating the floral bud physiological differentiation. Importantly, LcARR11-LcIPT3 may form a positive feedback regulatory loop that fosters the floral buds physiological differentiation in litchi.

In conclusion, our research reveals that during the physiological differentiation phase of litchi floral buds, the expression of *LcIPT3* is triggered by as-yet-unidentified factors, which in turn enhances cytokinin biosynthesis and leads to the activation of the transcription factor LcARR11. Subsequently, LcARR11 directly stimulates the expression of *LcFT1*, thereby promoting the physiological differentiation of floral buds. Notably, LcARR11 and *LcIPT3* might establish a positive feedback regulatory loop that fosters flowering in litchi. Our study offer valuable evidence for elucidating the molecular mechanisms by which cytokinins regulate floral bud differentiation in plants (Fig. 8).

## Materials and methods

### Plant materials and treatment

The ‘alternate-fruiting management’ was applied in Weiji Orchard in Dongguan City, Guangdong (22.98°N, 113.75°E). The orchard covers an area of 100 acres and is planted with the litchi variety ‘Xianjinfeng’. The orchard was divided into two zones: one designated as the on-year zone and the other as the off-year zone. These two zones switch to each other every year. For this study, in 2020, the trees in the off-year zone underwent girdling on their trunks when the last flush of autumn shoots matured. This procedure was intended to inhibit vegetative growth, thereby promoting normal floral bud differentiation and subsequent fruiting in 2021. As a result, the off-year trees in 2020 became on-year trees in 2021. In contrast, in 2020, the trees in the on-year zone were not girdled. Instead, they underwent heavy pruning just as they entered the stage of floral bud physiological differentiation. During this stage, upright or excessively dense large branches were removed from the base of the trees to stimulate vegetative growth and prevent the normal completion of floral bud differentiation. Consequently, the on-year trees in 2020 became off-year trees in 2021.

In 2021 and 2022, 200 trees from each of the on-year and off-year zones were randomly selected to investigate the flowering rate. Additionally, in 2021, five trees from each zone were randomly selected to examine the cytokinin content and gene expression patterns. Sampling of mature leaves from the last flush of autumn shoots began before the onset of low temperatures, with collections every two weeks. As the ‘whitish millet’ stage approached, the sampling frequency was increased to once per week until the appearance of ‘whitish millet,’ marking the completion of floral bud physiological differentiation in litchi.

For exogenous 6-BA treatment, in December 2022, nine uniformly conditioned off-year trees were randomly selected. Among these trees, three trees were treated with 20 mg/kg 6-BA, another three with 40 mg/kg 6-BA, while control groups received water-only treatments. 6-BA powder was first dissolved in a 1 mol/l NaOH solution to prepare a stock solution, and then was diluted in water for foliar spraying. The ‘kg’ refers to the weight of water used. Spraying commenced on 10 December 2022 (the arrival of low temperature), and was repeated every 15 days for a total of three applications. Leaf samples from the last flush of autumn shoots were collected at 0 DAT, 15 DAT, 30 DAT, 35 DAT, and 50 DAT (when ‘whitish millet’ appeared). To ensure the stability of endogenous cytokinins and accurate gene expression analysis, samples were flash-frozen in liquid nitrogen and maintained at −80°C pending subsequent analysis.

### Quantification of the percentage of flowering shoot

In March 2021 and March 2022, 200 trees were randomly selected from each of the on-year and off-year zones to investigate the flowering rate. For each tree, we quantified both the number of flowering shoot and the total potential fruiting shoot from four directions (east, south, west, north). The proportion of flowering shoots relative to the total number of potential fruiting shoots represented the percentage of flowering shoot for each tree. Subsequently, the percentage of flowering shoot of the trees were categorized into three groups: less than 30%, 40% to 70%, and greater than 80% to analyze the distribution pattern of the percentage of flowering shoots. In March 2023, the percentage of flowering shoot for each tree after 6-BA treatment were quantified.

### RNA-seq

Leaf total RNA was isolated employing Huayueyang Biotechnology's Ultra-Rapid Plant RNA Extraction System (Beijing, China). RNA quality was verified through spectrophotometric quantification (NanoDrop 2000) and electrophoretic separation on agarose gels. RNA samples were sent to Biomarker Technologies Co., Ltd for RNA-seq. After quality control checks, cDNA libraries were created and then processed on the Illumina platform. The RNA-seq data were analyzed using the BMKCloud platform (www.biocloud.net) and the bioinformatics tool TBtools [57]. Comparing the RNA expression levels in leaf samples from on-year trees and off-year trees at the same period, transcripts meeting the threshold criteria of FDR-adjusted *P* value <0.01 and minimum 2-fold expression variation were classified as differentially expressed.

### RT-qPCR analysis

Total RNA (1 μg) was reverse transcribed with TransScript® gDNA removal and cDNA synthesis reagents (TransGen Biotech). Quantitative PCR analysis was performed employing Hieff® qPCR SYBR® Green Master Mix (YEASEN) on a CFX96 platform (Bio-Rad). The expression levels were standardized using the reference genes *LcEF-1α* (litchi) or *AtUBQ* (Arabidopsis), with calculations performed employing the 2^−△△CT^ method [58, 59]. The primers used were listed in Table S7.

### WGCNA and KEGG pathway analysis

The WGCNA Shiny plugin tool in TBtools was used for the analysis [57]. The power value was set to 8 to ensure that the scale-free network topology structure R^2^ was greater than 0.8 and the mean connectivity dropped below 100. Gene modules were constructed by setting the module cuttree height to 0.35 and the minimum module size to 50. The constructed gene modules were then correlated with phenotype data (relative expression levels of *LcFT1*) to identify modules highly associated with the expression trend of *LcFT1*. A positive relationship is indicated by a number greater than 0, while a negative correlation is represented by a number less than 0. The greater the absolute value, the stronger the correlation. Genes from the identified module were extracted and subjected to KEGG pathway analysis using TBtools.

### Determination of endogenous cytokinin content

The content of endogenous cytokinins in litchi leaves was determined using UPLC-MS/MS [60]. A 0.25-g sample of litchi leaves was subjected to an 80:20 (v/v) mixture of acetonitrile and water for 8 h. The extract was purified using a Bond Elut Plexa PCX solid-phase extraction column and eluted with 2.5% ammonium methanol. The eluate underwent filtration through a 0.22-μm organic filter membrane for detection. A XSelect HSS T3 chromatographic column was used, and the mobile phase consisted of methanol and 5 mmol/l ammonium formate water solution. Gradient elution was performed for 7 min. Positive ion mode electrospray ionization (ESI+) was employed for sample ionization, and cytokinins were quantified using multiple reaction monitoring (MRM) mode.

### Subcellular localization analysis

The LcARR11 coding sequence was directionally cloned into pEAQ-GFP to create a C-terminal GFP fusion. *Agrobacterium* GV3101 harboring either pEAQ-LcARR11-GFP or the empty vector control was co-injected with NLS-mCherry into tobacco leaves. Following 48-h incubation, dual-channel fluorescence imaging was performed using a ZEISS LSM 7 DUO confocal system (excitation 488 nm for GFP, 561 nm for mCherry). The primers used were listed in Table S7.

### Electrophoretic mobility shift assay

PCR amplification of the LcARR11 DNA-binding domain coding sequence was followed by insertion into the pGEX-4 T-1 expression vector. Following transformation into E. coli BM Rosetta (DE3) cells, recombinant GST-fused protein expression was induced by 1 mM IPTG at 16°C for 16 h. The GST-LcARR11 fusion protein was subsequently affinity-purified using glutathione-agarose beads (YEASEN, Shanghai). Biotin-labeled probes containing binding sites (AGATT) derived from the *LcFT1* and *LcIPT3* promoter regions were generated, with the labeling at the 3′ end. Competition assays employed unlabeled wild-type and mutant oligonucleotides identical to the biotinylated probes. DNA-protein binding reactions containing *LcFT1* or *LcIPT3* probes and purified GST-LcARR11 were performed using the LightShift™ EMSA system (ThermoFisher), respectively. Images were captured using the ChemiDoc MP Imaging System (Bio-Rad, Hercules, CA, USA). The primers used were listed in Table S7.

### Dual-luciferase reporter assay

To test the transcriptional activity of LcARR11, the full-length CDS of *LcARR11* was subcloned into the pBD vector. The pBD and pBD-LcARR11 plasmids were used as Effectors, while the CaMV35S promoter-driven REN and LUC were used as Reporters. These plasmids were separately transformed into the *Agrobacterium strain* EHA105. The Effector and Reporter suspensions were mixed at a ratio of 9:1 and incubated for 3 h before being infiltrated into tobacco leaves. Following an incubation period of 48 to 72 h, the luciferase activity of LUC and REN was measured using the Dual-LUC assay reagents (YEASEN, Shanghai).

To further verify the interaction between LcARR11 and its downstream target genes *LcFT1* and *LcIPT3*, the coding sequence of LcARR11 was inserted into pGreenII 62-SK to create the effector construct, while *LcFT1* and *LcIPT3* promoter regions were engineered into pGreenII 0800-LUC as reporter plasmids. These constructs were introduced into *Agrobacterium* EHA105 (pSoup) for transient expression in tobacco leaves. Following 48 to 72 h of incubation, luminescence signals were quantified by measuring both firefly (LUC) and Renilla (REN) luciferase activities, with results expressed as LUC/REN ratios. Six biological replicates were performed for statistical validation. The primers used were listed in Table S7.

### Generation of transgenic *Arabidopsis*

The CDS of *LcARR11* and *LcIPT3* were cloned and ligated into the pCAMBIA1302 vector, respectively. The plant expression vectors containing *LcARR11* and *LcIPT3* under the CaMV 35S promoter were transformed into GV3101 competent cells for subsequent *Arabidopsis* floral dip transformation [61]. After obtaining T1 generation transgenic seeds, hygromycin-resistant plants were selected to obtain positive transgenic plants. Homozygous plants were obtained by self-pollination and used for flowering phenotype observation. The primers used were listed in Table S7.

### Data analysis

Results represent mean values derived from three to six biological replicates. Statistical comparisons between groups were performed using either Student’s *t*-test or Duncan’s multiple range test. The primers employed in this study are provided in Table S7.

## Supplementary Material

Web_Material_uhaf218

## Data Availability

All data generated during the study are provided in the text and supplementary files. And The RNA-seq data used in this study have been deposited in the National Center for Biotechnology Information (NCBI) BioProject database under accession number PRJNA1290538.
