# Supplementary material for: Cytokinin response factor LcARR11 promotes floral bud physiological differentiation by activating LcIPT3 and LcFT1 in litchi
Source: Hortic Res. 2025 Aug 26;12(11):uhaf218. doi: 10.1093/hr/uhaf218 (PMC12596081; doi:10.1093/hr/uhaf218)
Supplement: Web_Material_uhaf218 [file web_material_uhaf218.zip › Supplementary Figure.docx]

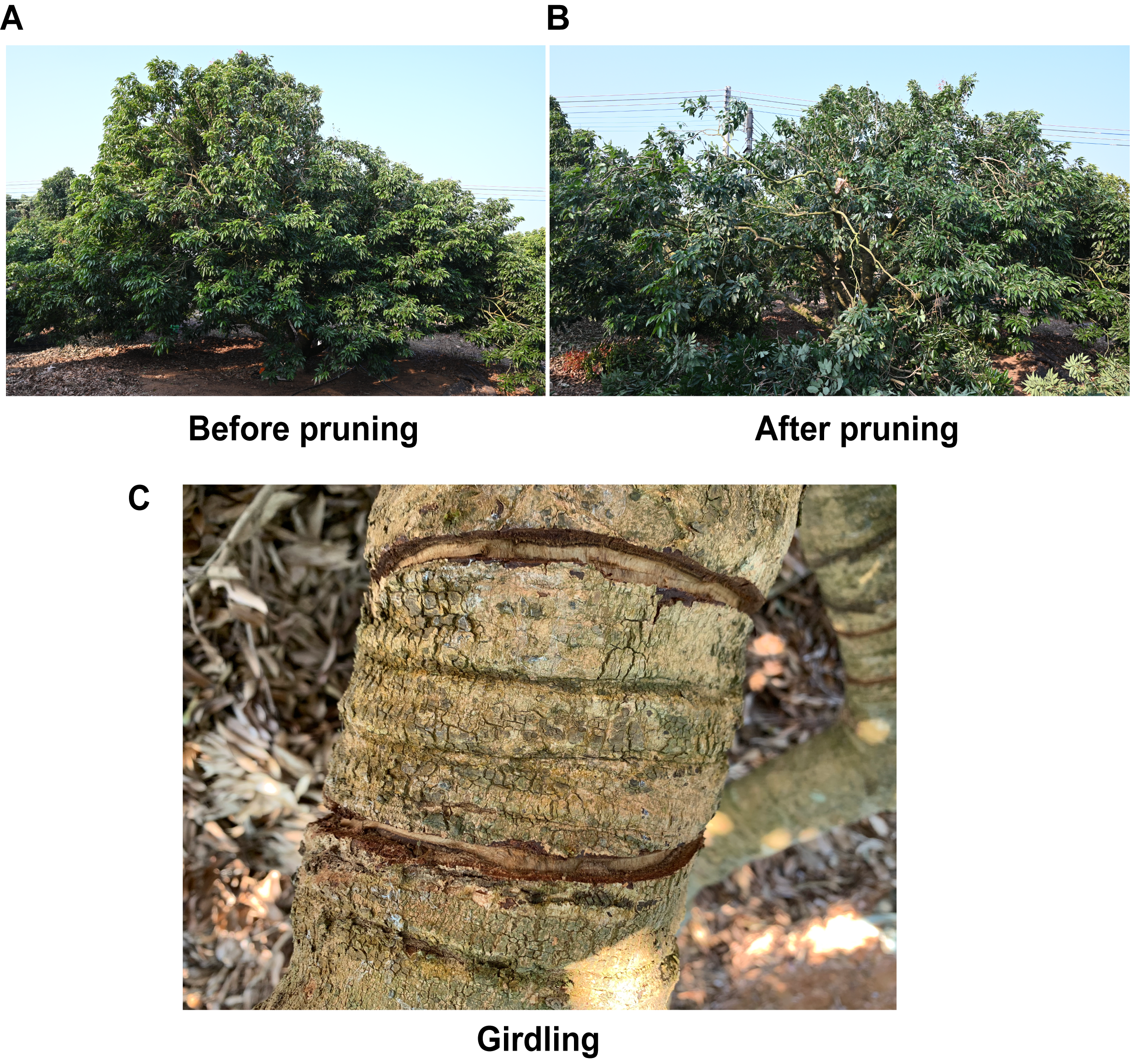


**Figure S1. Diagram of pruning for Off-year trees and girdling for on-year Trees. (A)** Status of off-year trees before pruning. **(B)** Status of off-year trees after pruning. After pruning, the vegetative growth of the tree was promoted, while its reproductive growth was suppressed, preventing it from completing flower bud differentiation normally, ultimately resulting in an off-year tree. **(C)** Status of on-year trees after girdling. After heavy girdling, the vegetative growth of the tree was suppressed, while its reproductive growth was promoted, allowing it to complete flower bud differentiation normally during winter, ultimately resulting in an on-year tree.


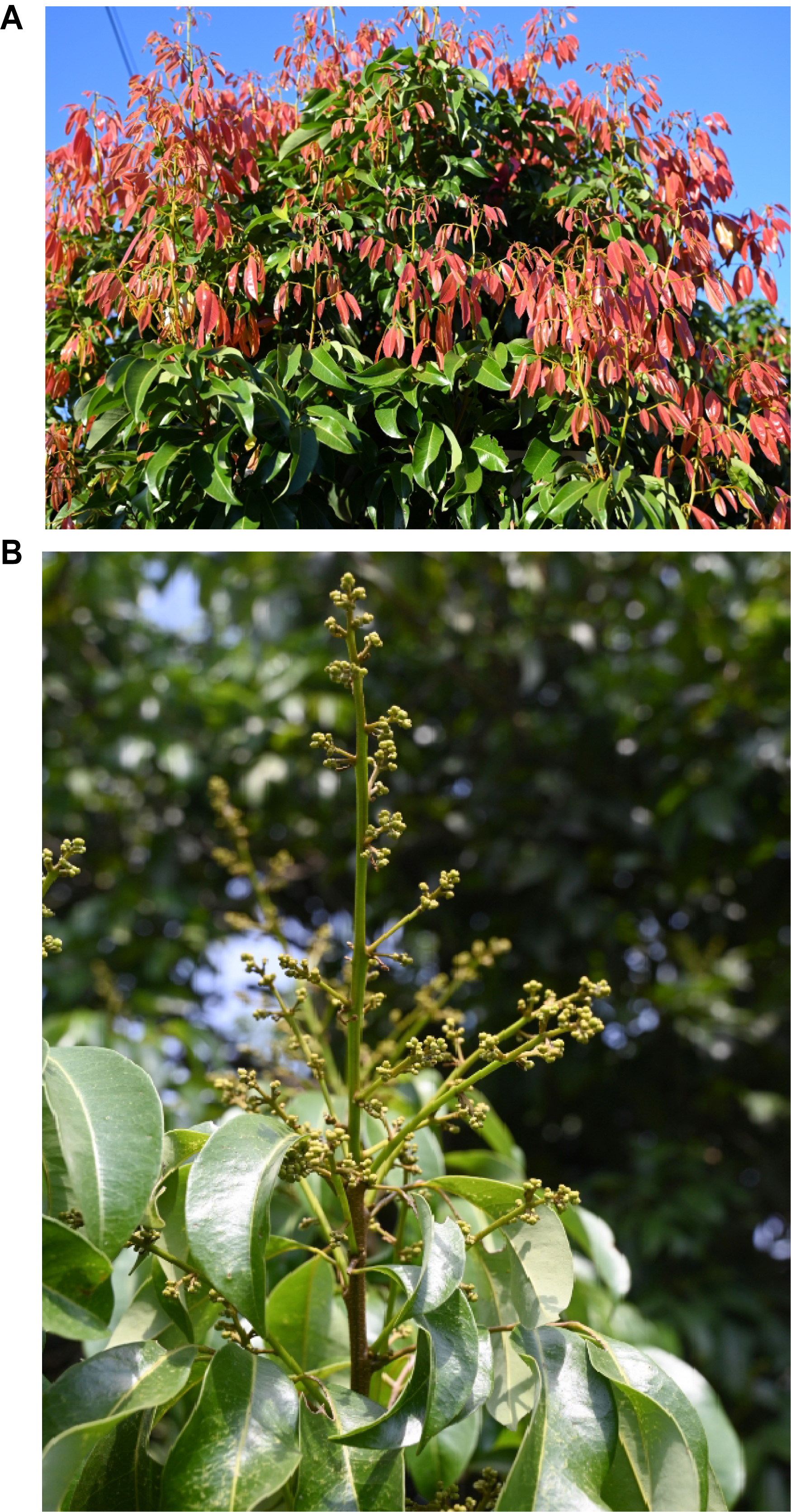


**Figure S2. Diagram of new shoots and inflorescence in litchi. (A)** During vegetative growth, new shoots emerge with red-colored, immature leaves. **(B)** Schematic illustration of a single inflorescence in litchi.


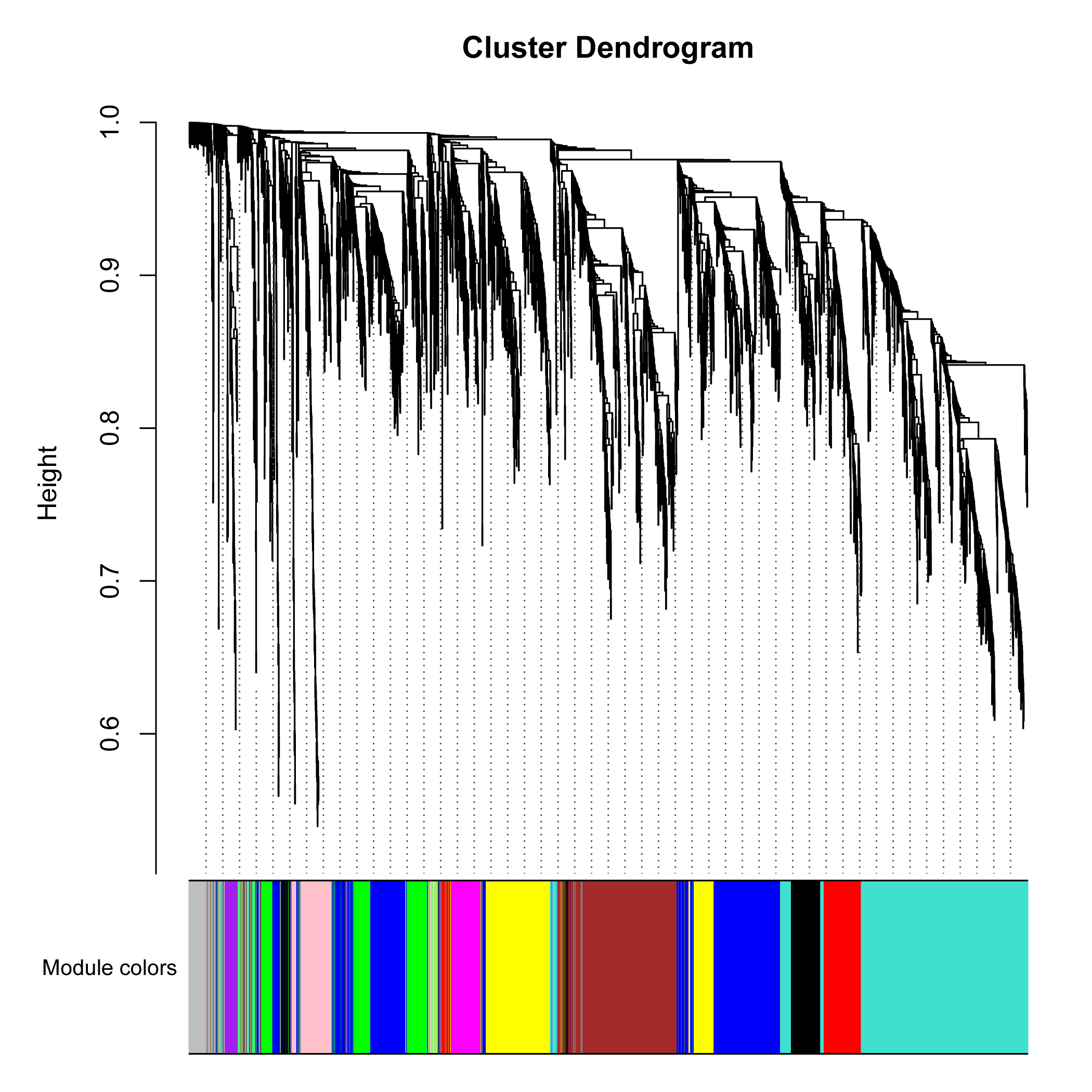


**Figure S3. The systematic clustering tree of differentially expressed genes based on TOM (Topological Overlap Matrix).** Each branch on the tree represents a gene, and different colors represent different co-expression modules. There are ten different modules in the clustering tree.


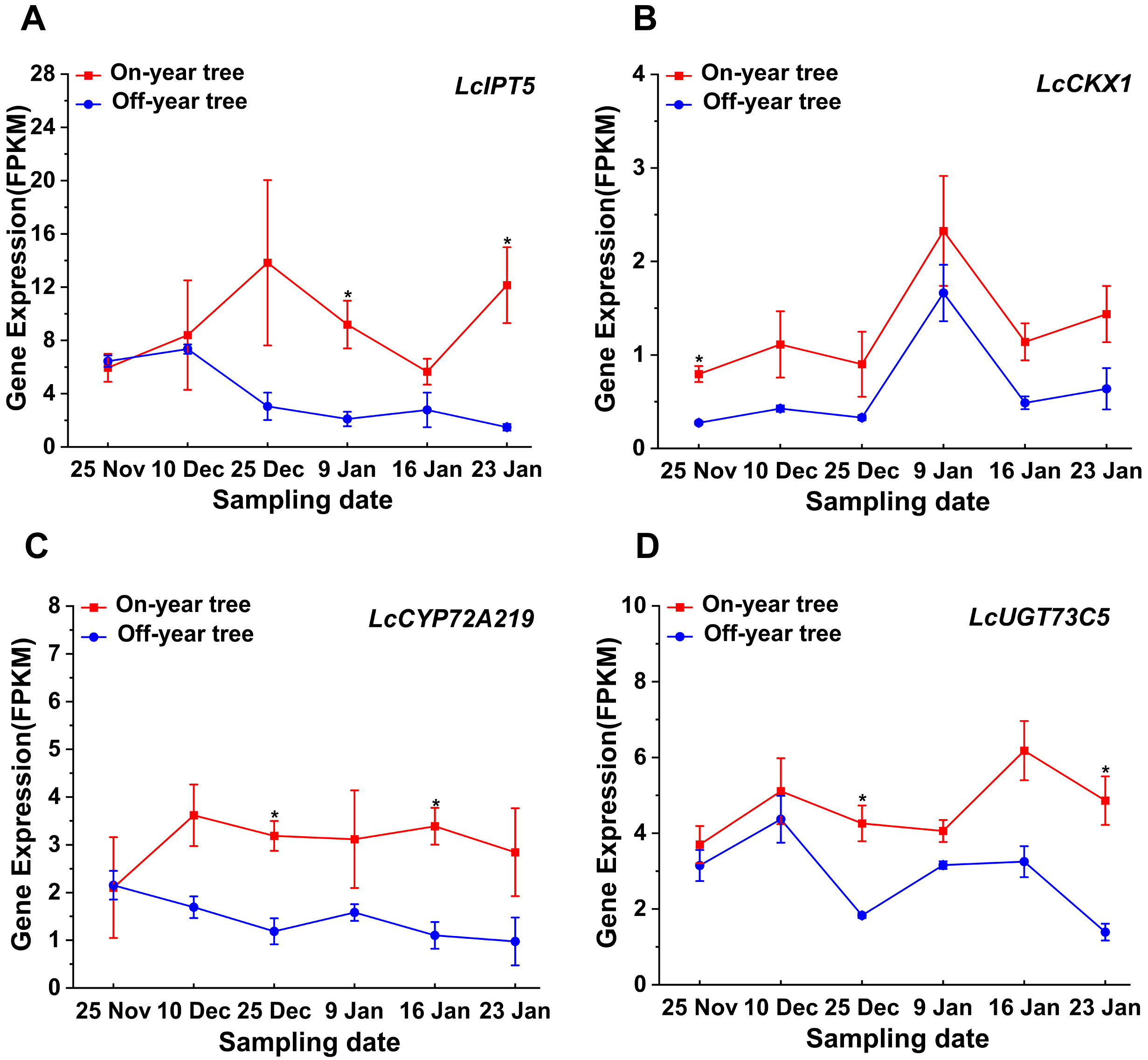


**Figure S4. The expression patterns of four genes in the zeatin biosynthesis pathway. (A)** The FPKM values (data from RNA-seq) of *LcIPT5* in leaves of on-year trees and off-year trees during the floral bud physiological differentiation in litchi. **(B)** The FPKM values (data from RNA-seq) of *LcCKX1* in leaves of on-year trees and off-year trees during the floral bud physiological differentiation in litchi. **(C)** The FPKM values (data from RNA-seq) of *LcCYP72A219* in leaves of on-year trees and off-year trees during the floral bud physiological differentiation in litchi. **(D)** The FPKM values (data from RNA-seq) of *LcUGT73C5* in leaves of on-year trees and off-year trees during the floral bud physiological differentiation in litchi.


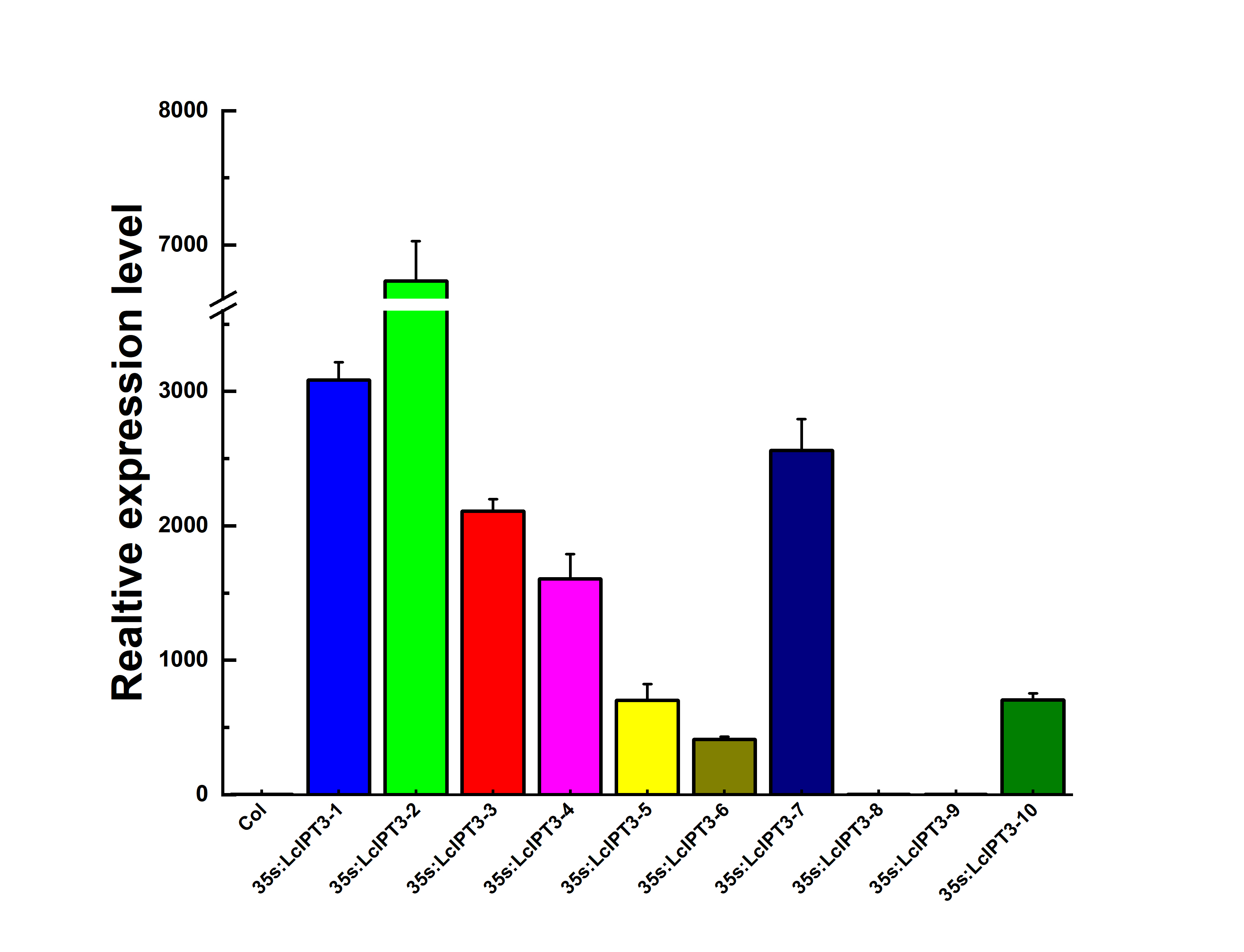


**Figure S5. The expression levels of *LcIPT3* in different transgenic *Arabidopsis* plants.** *AtUBQ* gene was used as the internal reference gene to calculate the expression levels of *LcIPT3* in the transgenic lines and wild-type plants. The expression level in the wild-type plants was set to 1, and the relative expression levels in the transgenic plants were determined accordingly. Each sample included three technical replicates.


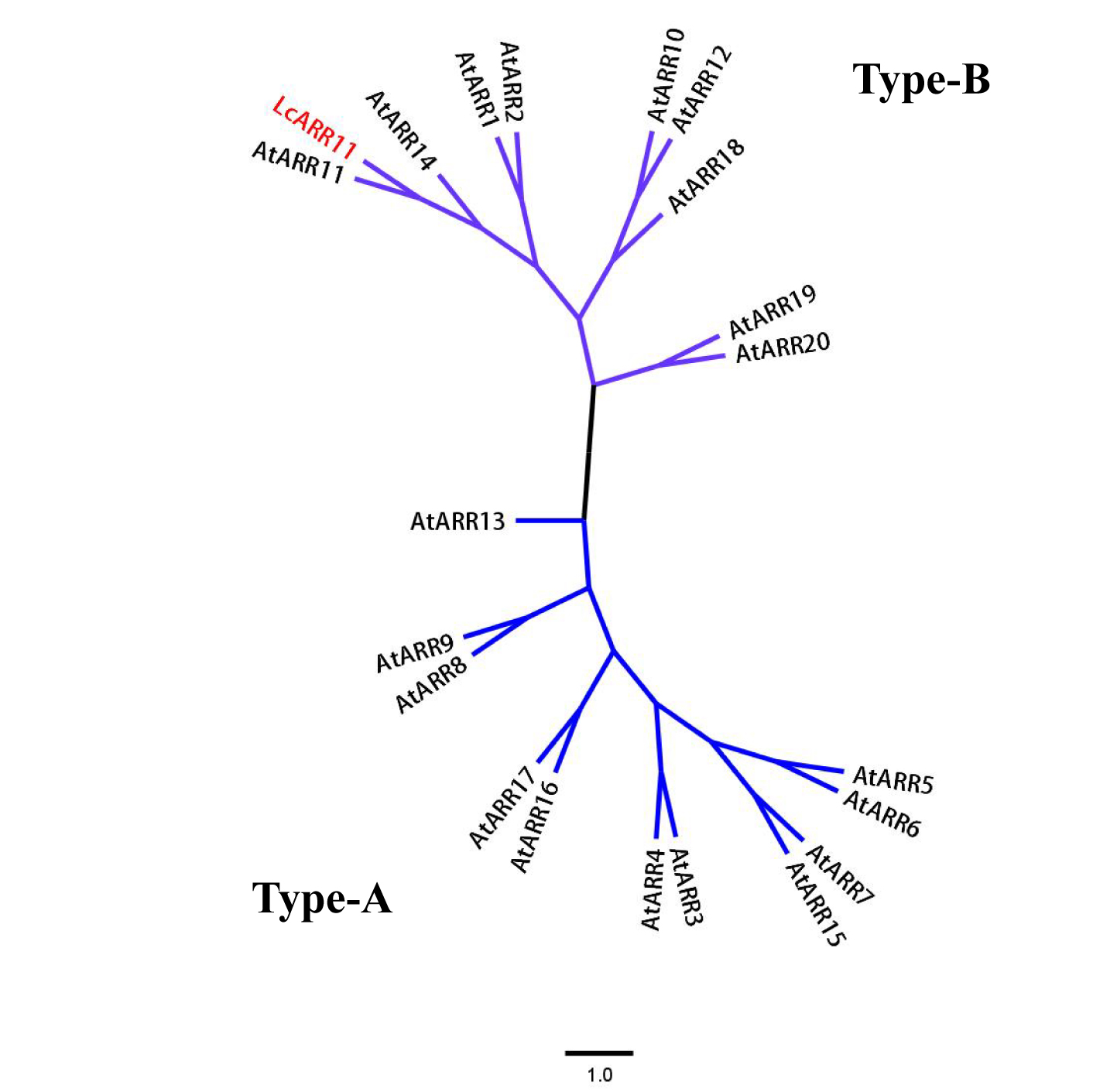


**Figure S6. The phylogenetic analysis of LcARR11 protein in litchi and *Arabidopsis.*** Phylogenetic tree showing the evolutionary relationships of LcARR11 protein from litchi compared to homologous proteins in *Arabidopsis* thaliana. The tree was constructed using the Neighbor-Joining method based on the amino acid sequences.


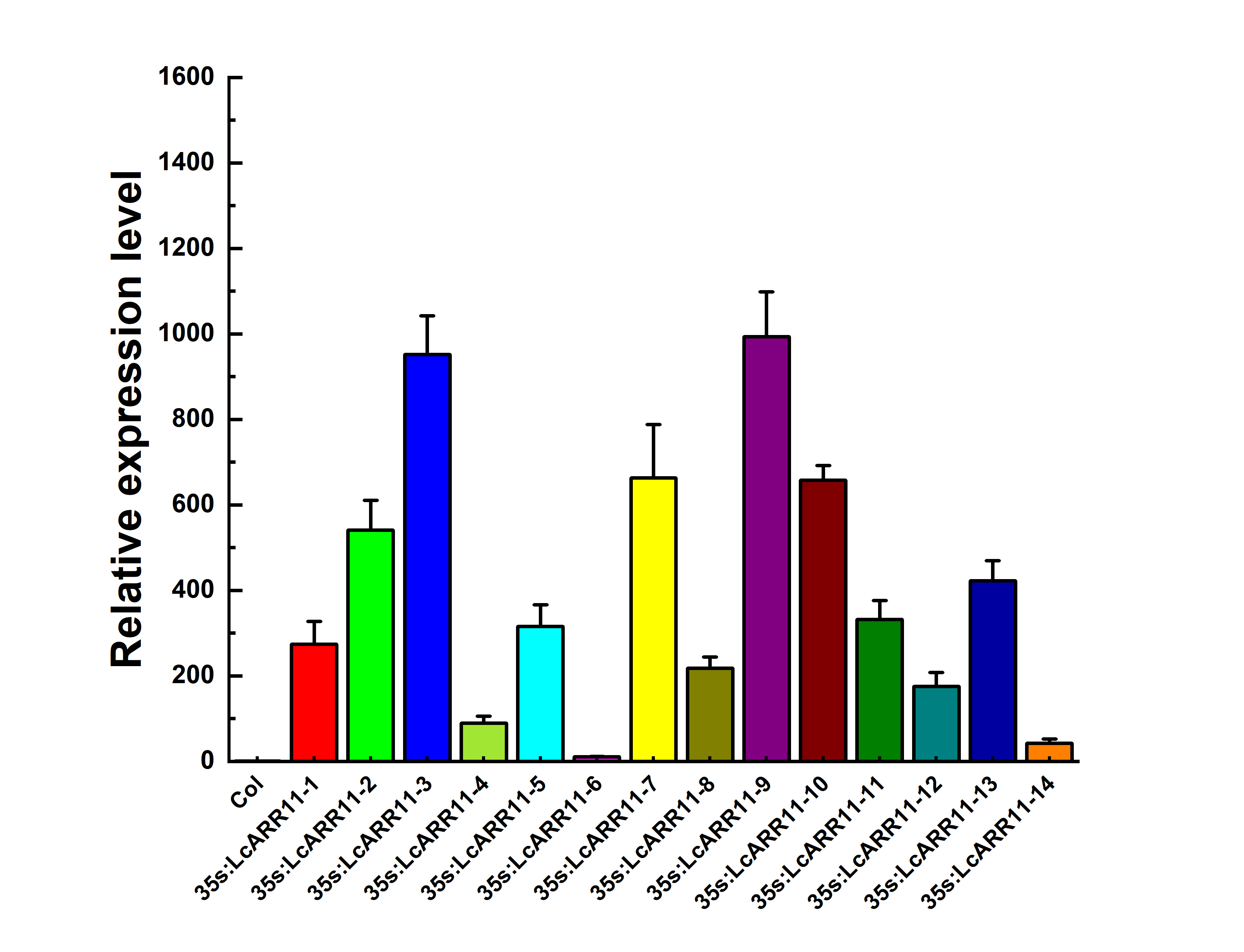


**Figure S7. The expression levels of *LcARR11* in different transgenic *Arabidopsis* plants.** *AtUBQ* gene was used as the internal reference gene to calculate the expression levels of *LcARR11* in the transgenic lines and wild-type plants. The expression level in the wild-type plants was set to 1, and the relative expression levels in the transgenic plants were determined accordingly. Each sample included three technical replicates.
